# Use of the Robson classification to understand the increased risk of cesarean section in case of maternal obesity

**DOI:** 10.1186/s12884-020-03410-z

**Published:** 2020-11-26

**Authors:** Simon Crequit, Diane Korb, Cécile Morin, Thomas Schmitz, Olivier Sibony

**Affiliations:** 1 Department of Gynecology and Obstetrics, Robert Debré University Hospital, AP-HP, 48 boulevard Sérurier, 75019 Paris, France; 2 Centre for Epidemiology and Statistics Sorbonne Paris Cité (CRESS), Obstetrical Perinatal and Pediatric Epidemiology Research Team, EPOPé, INSERM, INRA, Université de Paris, Paris, France

**Keywords:** Maternal obesity, Caesarean section, Robson, BMI and cesarean

## Abstract

**Background:**

The aim of this study was to identify characteristics of pregnant women with obesity that contribute to increased cesarean rate.

**Methods:**

Retrospective cohort in a single academic institution between 2012 and 2019. Women who delivered during this period were classified according to the Robson classification. Women with normal body mass index (*N* = 11,797) and with obesity (*N* = 2991) were compared. The contribution of each Robson group to the overall caesarean rate were compared.

**Results:**

The overall cesarean rate was higher for women with (28.1%) than without (14.2%, *p* < 0.001) obesity. This result came mainly from Robson group 5a (history of one cesarean). After adjustment for medical factors within this group, the association between maternal obesity and cesarean during labor was significant.

**Conclusions:**

The higher cesarean rate in women with obesity is explained by Robson group 5a in which obesity is an independent risk factor of in labor cesarean delivery.

**Supplementary Information:**

The online version contains supplementary material available at 10.1186/s12884-020-03410-z.

## Background

Maternal obesity is a major health issue in most of industrialized countries. The rate of obesity among pregnant women has increased from 9.9 to 11.8% between 2010 [1] and 2016 [2] in France and from 17.6% in 2003 to 24.5% in 2014 in the US [3, 4]. Previous studies on women with obesity have demonstrated increased pregnancy complications such as hypertensive disorders, gestational diabetes, macrosomia and stillbirth [5–7]. Recent data on maternal obesity have demonstrated an increase in cesarean section (CS) and an increasing number of elective cesarean delivery [8*–*10]*.* Indeed, the CS rate increase in women with obesity is becoming of concern given the frequency of this disorder. Unfortunately, these data are often focusing on a small part of the pregnant population with obesity such as primiparas, women developing gestational diabetes or only focus on complications. Yet, there are no available data on the risk of caesarean delivery in this population aiming at identifying subgroups at high risk of CS in order to implement measures to reduce the CS rate. To address this issue the Robson classification [11] constitutes a useful tool to identify the characteristics of women contributing the most to the CS rate in a given population. The latter divides births into 10 groups based on obstetrical history, onset of labor, fetal presentation, number of neonates, and gestational age. It is a standardized and reproducible framework that classifies women in relevant categories for analysis of CS rates.

The aim of this study was to identify the characteristics of the women with obesity that contribute to the overall cesarean rate increase using the Robson classification. The objective was to compare the contribution of each Robson group to the overall cesarean rate between normally weighted and women with obesity to target the group responsible for most of the difference in CS rate. This analysis might indicate in which group efforts have to be made in order to reduce the overall CS rate in women with obesity.

## Methods

### Study population

**Fig. 1:**
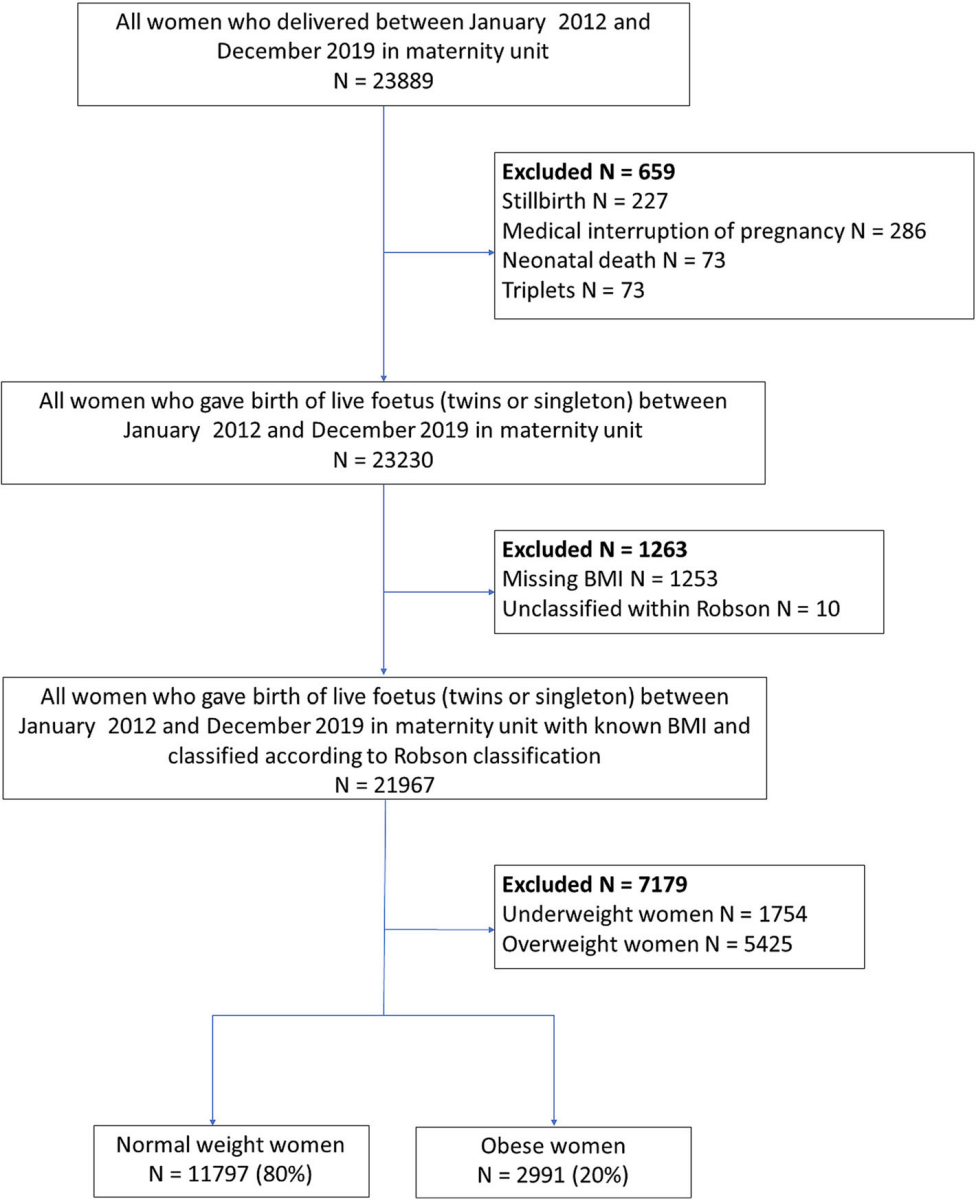
Flow chart

Using hospital birth records, we identified all women that delivered at a single tertiary care academic institution between January 2012 and December 2019. Stillbirths and neonatal deaths that might modify the mode of delivery were excluded. Triplets (rare event, non-consensual management), unknown pre-pregnancy body mass index (BMI), according to the World Health Organization’s (WHO) definition [12] and patients who couldn’t be classified within the Robson classification were excluded (Fig. 1: flow chart). Underweight women (BMI < 18.5 kg/m^2^) and overweight women (BMI [25–29.9] kg/m^2^) were excluded because this study focuses on the effect of maternal obesity for which normally weighted women represents the best comparison group. We compared women with obesity (BMI ≥ 30 kg/m^2^) to normally weighted women (BMI [18.5–24.9] kg/m^2^).

### Collected data

Maternal weight was recorded at each prenatal care visit. Total gestational weight gain (GWG) was calculated by subtracting pre-pregnancy weight from the last recorded weight before delivery. Social and demographic characteristics, pregnancy characteristics, labor, maternal and neonatal outcomes were collected from medical records.

The entire medical file was computerized and was completed prospectively at each visit, at delivery, and during the post-partum period by health professional including midwives and obstetrician in charge of the patient. All data were verified at the daily obstetrical staff meeting. During the study period, all women were managed regardless of their BMI regarding the choice of the mode of delivery, induction of labor, and labor management. Gestational diabetes screening was done according to the French recommendation [13].

This information was used to classify women into the 10 Robson groups according to their BMI. All women were classified according to the Robson classification based on obstetrical history, onset of labor, fetal lie, number of fetuses, and gestational age. The analysis was performed using the Robson classification with subdivision in which groups 2 and 4 are separated in cesarean during labor (groups 2a and 4a) and cesarean before labor (groups 2b and 4b). Within Group 5, this classification separate women with one previous cesarean delivery (5a) from women with more than one previous cesarean (5b) because a history of two or more CS is usually considered as an indication for elective cesarean delivery. Moreover, in the maternity at study a history of one CS was not an indication for repeated cesarean delivery. Groups 1 to 4 are usually called low risk groups in terms of cesarean section as they include women delivering at term with a singleton fetus in cephalic presentation whereas groups 5 to 10 are defined as high risk groups of cesarian (history of CS, breech, multiple pregnancy and premature birth).

### Outcomes: cesarean delivery rates

The first analysis consisted in calculating the overall CS rates within the two groups. We then tested if the distribution of patients among the Robson classification differed between the two studied groups. For each Robson group we calculated the relative size of the group (number of deliveries within the group/all deliveries in the BMI group), the CS rate within the group (number of CS within the group/total deliveries within the group) and the contribution of the group to the overall CS rate (CS in each group/total deliveries in the BMI group).

### Statistical analysis

We compared the characteristics of the women, pregnancies, labors and neonates, according to maternal pre-pregnancy BMI, based on Chi^2^ or Fisher exact tests for categorical variables and Student’s or Wilcoxon rank sum tests for quantitative variables, as appropriate. All tests were two-sided with *P*-values ≤.05 defined as statistically significant. Within the Robson categories, analyses were performed for cesarean delivery before labor (vs during labor) and intrapartum cesarean (vs vaginal delivery) among women who labored. Multivariate logistic regression models were created to assess the direct effect of maternal pre-pregnancy BMI on cesarean delivery. The variables introduced into the models were those clinically relevant or found in the literature [14]. Because some of the explaining variables were highly correlated with maternal obesity, the models were tested for multicollinearity. R software (R Development Core Team (2008), version 3.4.4) was used for all analyses.

## Results

Among the 14,788 women included in this analysis, 11,797 (79.8%) had a normal BMI, and 2991 (20.2%) had a BMI ≥30 (Fig. 1). Within the group of women with a BMI ≥30, 2149 (71.8%) were grade 1 obesity (BMI [30–35[kg/m^2^), 628 (20.9%) were grade 2 (BMI [35–40[kg/m^2^) and 214 (7.1%) were grade 3 (BMI > 40 kg/m^2^).

Women with obesity were more often older than 35 compared to women with normal BMI. Women with BMI ≥30 had more often history of chronic hypertension, diabetes mellitus and were more often multiparous with a history of previous CS than women with normal BMI. Moreover, women with obesity presented higher medical risk level at the beginning of pregnancy compared to normally weighted women. Complications of pregnancy were more frequent for women with obesity compared to normal weight women with significantly more gestational hypertensive disorders (gestational hypertension and preeclampsia) and gestational diabetes (requiring insulin or simple diet). The maternal obesity group significantly exceeded the total GWG recommended in guidelines. Women with a BMI ≥ 30 displayed more premature birth before 34 weeks of gestation compared to normally weighted women. Induction was more frequent for women with obesity. Moreover, the induction rates were higher for hypertensive gestational pathologies, fetal indication or maternal indication compared to normal weight women (Table 1).

**Table 1:** Characteristics of women according to pre-pregnancy BMI

	Normal weight	Obesity	*p*
	11,797	2991	
	N (%)	N (%)	
**Maternal characteristics**			
Age (years) (mean ± sd)	31.82 ± 5.51	32.88 ± 5.45	< 0.001
Age class (years)			< 0.001
< 25	1343 (11.4)	213 (7.1)	
[25–30[	3096 (26.2)	735 (24.6)	
[30–35[	3953 (33.5)	974 (32.6)	
≥ 35	3405 (28.9)	1069 (35.7)	
Multiparous women	6639 (56.3)	2280 (76.2)	< 0.001
Previous cesarean delivery			< 0.001
1	1082 (9.2)	561 (18.8)	
≥ 2	220 (1.9)	246 (8.2)	
Body mass index before pregnancy (kg/m^2^) (mean ± sd)	21.48 ± 1.66	33.47 ± 3.54	< 0.001
Smoker	1585 (13.7)	307 (10.6)	< 0.001
Diabetes mellitus	94 (0.8)	113 (3.8)	< 0.001
Chronic hypertension	116 (1.0)	163 (5.4)	< 0.001
Bariatric surgery			< 0.001
Bypass	30 (0.3)	36 (1.2)	
Sleeve gastrectomy	28 (0.2)	56 (1.9)	
Gastric band	14 (0.1)	68 (2.3)	
Hight medical risk level at the beginning of pregnancy^a^	1880 (15.9)	878 (29.4)	< 0.001
**Pregnancy characteristics**			
Complications of pregnancy^b^	1042 (8.8)	870 (29.1)	< 0.001
Twin pregnancy	481 (4.1)	105 (3.5)	0.172
Fetal presentation			0.019
Cephalic	11,117 (94.2)	2782 (93.0)	
Breech	659 (5.6)	198 (6.6)	
Transverse	20 (0.2)	10 (0.3)	
Weight intake during pregnancy (mean ± sd)	12.24 ± 5.28	7.74 ± 7.00	< 0.001
Excessive total GWG^c^	2691 (23.7)	1308 (45.7)	< 0.001
Gestational diabetes requiring insulin	162 (1.4)	295 (9.9)	< 0.001
Gestational diabetes without insulin	477 (4.0)	361 (12.1)	< 0.001
In utero transfer	198 (1.7)	57 (1.9)	0.439
Premature rupture of membranes	163 (1.4)	76 (2.5)	< 0.001
Preterm labor	725 (6.1)	158 (5.3)	0.083
Gestational hypertension	80 (0.7)	105 (3.5)	< 0.001
Preeclampsia	233 (2.0)	151 (5.0)	< 0.001
HELLP syndrome	15 (0.1)	8 (0.3)	0.081
Eclampsia	2 (0.0)	1 (0.0)	0.57
Suspected small for gestational age	254 (2.2)	40 (1.3)	0.005
Cholestasis	126 (1.1)	40 (1.3)	0.25
Deep vein thrombosis during pregnancy	9 (0.1)	3 (0.1)	0.958
Pulmonary embolism during pregnancy	1 (0.0)	3 (0.1)	0.035
**Delivery characteristics**			
Gestational age at delivery			
< 28	70 (0.6)	33 (1.1)	0.004
[28–34[	304 (2.6)	106 (3.5)	0.004
[34–37[	679 (5.8)	186 (6.2)	0.35
[37–41[	8634 (73.2)	2124 (71.0)	0.02
≥ 41	2110 (17.9)	542 (18.1)	0.78
Induction	2515 (21.3)	870 (29.1)	< 0.001
Induction indication			< 0.001
Fetal	826 (7.0)	258 (8.6)	0.003
Maternal	155 (1.3)	82 (2.7)	< 0.001
Premature rupture of membranes	857 (7.3)	251 (8.4)	0.04
Post term	404 (3.4)	131 (4.4)	0.01
Gestational hypertension or preeclampsia	246 (2.1)	146 (4.9)	< 0.001
Non medical	27 (0.2)	2 (0.1)	0.11
Delivery mode			< 0.001
Vaginal delivery	10,130 (85.9)	2150 (71.9)	
Cesarean delivery	1667 (14.1)	841 (28.1)	

^a^High medical risk level at the beginning of pregnancy was defined as the presence of one or more of: history of cardiac disease, hypertension, diabetes, venous thrombosis, pulmonary embolism, Graves’ disease, asthma, homozygous sickle cell anemia, thrombocytopenia, coagulation disorder, a rare or systemic disease, nephropathy, HIV infection, pre-eclampsia, growth restriction, preterm delivery, fetal death or neonatal death

^b^Defined as the occurrence of one or more of the following complications: gestational diabetes, pre-eclampsia, eclampsia, HELLP syndrome, venous thrombosis, pulmonary embolism, severe sepsis, convulsions, diabetic ketoacidosis, coagulation disorder, cholestasis of pregnancy

^c^Excessive total gestational weight gain defined as an intake of more than 9 kg for women with obesity and an intake of more than 15.9 kg for normal weight

The overall CS rates were significantly different between the two groups (28.1% for women with obesity versus 14.1% for normal weight women, *p* < 0.001, Table 1).

Concerning the distribution into the Robson classification, normal weight women were more often classified in groups 1 (Primiparous, single cephalic presentation, spontaneous labor, ≥ 37 weeks), 2 (Primiparous, single cephalic presentation, ≥ 37 weeks), 3 (Multiparous, single cephalic presentation, spontaneous labor, ≥ 37 weeks), and 6 (Primiparous, single breech presentation) (Tables 2, 3). Women with obesity were more often classified in group 4 (Multiparous, single cephalic presentation, ≥ 37 weeks, induced or CS before labor), 5 (Multiparous, single cephalic presentation, history of one or more CS, ≥ 37 weeks, induced or CS before labor), 7 (Multiparous, single breech presentation, including a history of CS) and 10 (Single cephalic presentation, < 37 weeks, including a history of one or more CS), (Tables 2, 3). No differences were found in group 8 (Twin pregnancies, including a history of one or more CS) and 9 (Single transverse or oblique lie, including a history of one or more CS) (Table 3).

**Table 2:** Cesarean delivery profiles of women with normal BMI using Robson classification

Group*	***N CS in group***	***Total N in group***	***Group Size (%) 1***	***Group CS rate (%) 2***	***Absolute group contribution to overall CS rate (%) 3***	***Relative group contribution to overall CS rate (%) 4***
***1***	281	3226	27.3	8.7	2.4	16.9
***2***	288	1130	9.6	25.5	2.4	17.3
*2a (Induced)*	234	1076	9.1	21.7	2.0	14.0
*2b (Prelabor CS)*	54	54	0.5	100.0	0.5	3.2
***3***	68	3943	33.4	1.7	0.6	4.1
***4***	51	735	6.2	6.9	0.4	3.1
*4a (Induced)*	31	715	6.1	4.3	0.3	1.9
*4b (Prelabor CS)*	20	20	0.2	100.0	0.2	1.2
***5***	360	1074	9.1	33.5	3.1	21.6
*5.a (1 CS)*	204	912	7.7	22.4	1.7	12.2
*5.b (> 1 CS)*	156	162	1.4	96.3	1.3	9.4
***6***	150	272	2.3	55.1	1.3	9.0
***7***	134	266	2.3	50.4	1.1	8.0
***8***	160	478	4.1	33.5	1.4	9.6
***9***	20	20	0.2	100.0	0.2	1.2
***10***	155	653	5.5	23.7	1.3	9.3
***Total***	1667	11,797	100.0	14.1	14.1	100.0

*Group 1: primiparous, single cephalic presentation, spontaneous labor, ≥ 37 weeks

*Group 2: primiparous, single cephalic presentation, ≥ 37 weeks

*Group 2a: primiparous, single cephalic presentation, ≥ 37 weeks, induction

*Group 2b: primiparous, single cephalic presentation, ≥ 37 weeks, CS before labor

*Group 3: multiparous, single cephalic presentation, spontaneous labor, ≥ 37 weeks

*Group 4: multiparous, single cephalic presentation, ≥ 37 weeks

*Group 4a: multiparous, single cephalic presentation, ≥ 37 weeks, induction

*Group 4b: multiparous, single cephalic presentation, ≥ 37 weeks, CS before labor

*Group 5: multiparous, single cephalic presentation, history of one or more CS, ≥ 37 weeks

*Group 5a: multiparous, single cephalic presentation, history of one or more CS, ≥ 37 weeks, history of one CS

*Group 5b: multiparous, single cephalic presentation, history of one or more CS, ≥ 37 weeks, history of more than one CS

*Group 6: primiparous, single breech presentation

*Group 7: multiparous, single breech presentation, including a history of CS

*Group 8: twin pregnancies, including a history of one or more CS

*Group 9: single transverse or oblique lie, including a history of one or more CS

*Group 10: single cephalic presentation, < 37 weeks, including a history of one or more CS

1. % = n of women in the group / total N women delivered in the setting × 100

2. % = n of CS in the group / total N of women in the group × 100

3. % = n of CS in the group / total N of women delivered in the setting × 100

4. % = n of CS in the group / total N of CS in the setting × 100

**Table 3:** Cesarean delivery profiles of women with obesity using Robson classification

Group*	***N CS in group***	***Total N in group***	***Group Size (%) 1***	***Group CS rate (%)2***	***Absolute group contribution to overall CS rate (%) 3***	***Relative group contribution to overall CS rate (%) 4***
***1***	66	346	11.6***	19.1***	2.2	7.8***
***2***	99	240	8.0**	41.3***	3.3**	11.8***
*2a (Induced)*	83	224	7.5**	37.1***	2.8**	9.9**
*2b (Prelabor CS)*	16	16	0.5	100.00%	0.5	1.9
***3***	43	910	30.4**	4.7***	1.4***	5.1
***4***	52	351	11.7***	14.8***	1.7***	6.2***
*4a (Induced)*	41	340	11.4***	12.1***	1.4***	4.9***
*4b (Prelabor CS)*	11	11	0.4	100	0.4	1.3
***5***	342	659	22.0***	51.9***	11.4***	40.7***
*5.a (1 CS)*	154	468	15.6***	32.9***	5.1***	18.3***
*5.b (> 1 CS)*	188	191	6.4***	98.4	6.3	22.4
***6***	17	29	1.0***	58.6	0.6**	2.0***
***7***	92	136	4.5***	67.6**	3.1***	10.9*
***8***	44	104	3.5	42.3	1.5	5.2***
***9***	10	10	0.3	100	0.3	1.2
***10***	76	206	6.9**	36.9***	2.5***	9
***Total***	841	2991	100	28.1***	28.1	100

* See groups description in Table 2

1. % = n of women in the group / total N women delivered in the setting × 100

2. % = n of CS in the group / total N of women in the group × 100

3. % = n of CS in the group / total N of women delivered in the setting × 100

4. % = n of CS in the group / total N of CS in the setting × 100

Comparisons using Chi square test with the normal weight groups, * *p* < 0.05, ** *p* < 0.01, *** *p* < 0.001

Cesarean delivery profiles of the normal weight and the maternal obesity groups are presented in Table 2 and Table 3, respectively. Within each group of the Robson classification women with obesity delivered more often by CS than normal weight women (Table 3). The CS rate between women with obesity and normally weighted women were significantly different for Robson group 1 (respectively 19.1% versus 8.7%, *p* < 0.001), Robson group 2a (41.3% versus 21.7%, *p* < 0.001), Robson group 3 (4.7% versus 1.7%, *p* < 0.001), Robson group 4a (12.1% versus 4.3%, *p* < 0.001), Robson group 5a (32.9% versus 22.4%, *p* < 0.001), Robson group 7 (67.6% versus 50.4%, *p* < 0.01) and Robson group 10 (36.9% versus 23.7%, *p* < 0.001).

The Robson category 5 (Multiparous, single cephalic presentation, history of one or more CS, ≥ 37 weeks) contributed the most to the difference in cesarean rates between normally weighted women and women with obesity with a difference in absolute contribution of 8.4%. This figure is due to a bigger size of the group and a higher CS rate for women with obesity. The overall cesarean rate in group 5a was increased for the women with obesity group compared to the normal weight group: 32.9 and 22.4% respectively (*p* < 0.001). The results were similar when we distinguished CS before labor (6.8% for women with obesity versus 4.8% for normal weight group, *p* < 0.001, Table S2) and CS during labor (26.1% for women with obesity versus 17.5% for normal weight group, *p* < 0.001, Table S2). Women with obesity had more cesarean section for abnormal fetal heart rate (13,7% versus 9.4% for normal weight women, *p* = 0.02) and arrest of labor (13.2% versus 8.3% for normal weight women, *p* = 0.005, Table S2). Induction rates were higher for women with obesity in group 5a (33,8% versus 22% for normally weighted women, *p* < 0.001, Table S2) especially for fetal, maternal and gestational hypertensive disorders.

After adjustment, the association between maternal pre-pregnancy BMI and cesarean delivery before labor in group 5a was not statistically significant (aOR = 1.26 CI: [0.76–2.08], adjustment for maternal age, high medical risk level at the beginning of pregnancy, pregnancy complications Table S3), whereas the association between maternal pre-pregnancy BMI and cesarean delivery during labor in group 5a was statistically significant (aOR =1.43, 95% CI: [1.07–1.9], adjustment for maternal age, high medical risk level at the beginning of pregnancy, pregnancy complication and induction, Table S4).

Neonates of women with obesity compared with the ones of normally weighted women had more often fetal macrosomia, presented more Apgar score < 7 at 5 min and more transfers in NICU or neonatal reanimation (Table 4, Table S5).

**Table 4:** neonatal outcomes of singleton pregnancies according to maternal pre-pregnancy BMI

	Normal weight	Obesity	p
	*N* = 11,316	*N* = 2886	
	*N* (%)	*N* (%)	
Birth weight (grams) (mean ± sd)	3233 ± 568	3313 ± 688	< 0.001
Birth weight (grams)			< 0.001
[2500–3800[	8981 (79.4)	2011 (69.7)	
< 2500	850 (7.5)	252 (8.7)	
> 3800	1485 (13.1)	623 (21.6)	
pH at ombilical cord			< 0.001
pH < 7	36 (0.3)	19 (0.7)	
pH [1, 7]	150 (1.4)	64 (2.3)	
pH]7.1–7.2[	709 (6.4)	179 (6.3)	
pH ≥ 7.2	10,185 (91.9)	2558 (90.7)	
Apgar score < 7 at 5 min	132 (1.2)	68 (2.4)	< 0.001
Neonatal transfer			< 0.001
Neonatal reanimation unit	443 (3.9)	175 (6.1)	
Intensive care unit	622 (5.5)	223 (7.8)	
Other specialized services	65 (0.6)	20 (0.7)	

## Discussion

### Main finding

Women with obesity are more likely to deliver by CS compared to normal weight women and this trend was similar in each of the Robson classification group. The increase in the overall CS rate in case of maternal obesity is mostly explained by women presenting a history of previous CS (Robson group 5a). After adjustment for pregnancy complications and medical factors in group 5a, the association between maternal obesity and CS before labor was not significant. However, maternal obesity remained an independent risk factor of in labor CS within this group.

### Interpretation

This work suggests that in order to reduce the overall CS rate in women presenting maternal obesity we should focus on women with a history of a previous CS (Robson group 5a). Among women of group 5a, this analysis shows that the risk of CS before labor could be explained by medical factors and complications of pregnancy. Indeed, women with obesity were more likely to present high medical risk level at the beginning of pregancy and pregnancy complications that are not included in the Robson classification. Yet these conditions are relevant to discuss the mode of delivery. In that case the Robson classification might be of limited value to reduce the number of CS performed in obese women. Therefore, a better management of the pre-existing complications and planning for prenatal care and delivery by the obstetrical team would improve obstetrical outcomes by reducing the impact of pregnancy complications and the requirement of repeated CS before labor [12]. Indeed, recent works on nutritional management of patients with weight excess demonstrated that maintaining or even reducing pre-pregnancy BMI can limit the occurrence of pregnancy complication thanks to better total GWG management [15, 16].

The increase of in labor CS for women with obesity within group 5a was not explained by medical factors. Yet, the increase of adverse pregnancy outcomes with maternal BMI increase results in a higher rate of induction [17]. It has been demonstrated that failed induction was more frequent among obese women and that its occurrence was parallel to maternal BMI increase [18]. This could explain in part the increased in labor CS rate observed for women with obesity.

Moreover, the increase of in labor CS for arrest of labor in women with obesity observed in our study is consistent with the literature. Indeed, women with obesity have been shown to present an altered first phase of labor and an increase in labor obstruction [19, 20]. This point might be due to a less effective myometrium in term of contractility [21] or a decrease in oxytocin receptors parallel to maternal BMI increase [22] and a higher blood rate of Leptin and cholesterol impairing myometrial contractility [23].

The main strength of this study is that it identified the characteristics of women with obesity responsible for the overall CS rate increase for the first time. A large number of women was included which allows to have a power necessary to highlight a difference between the maternal BMI groups if it exists. Attending practitioners prospectively collected the data about the management of the pregnancy, labor and delivery, and these data were ascertained routinely by medical staff the day after the delivery, so that thorough and accurate information was available for adjustment in the multiple logistic regression models. Moreover, the collection of induction indications and CS indications allowed a better understanding of the differences observed thanks to the Robson classification pertinence in the analyses of CS profiles.

The unicentric design of the study can limit the generalization of the results. Moreover, this study was conducted in an institution that present a lower CS rate compared to the average national rate [2] and all women who presented a history of one CS were encouraged to labor. Finally, few data about the characteristics of labor were analyzed including the cervical dilation at which the CS occurred.

Although, women with a BMI ≥30 have an increased rate of in labor CS, most of them undergo planned CS for a history of two or more CS. In order to reduce the CS rate in this population we should first improve prenatal care and nutritional management to avoid repeated CS before labor. Secondly, further studies must focus on the labor of women with a history of one CS (Robson group 5a) to identify risk factors of repeated CS.

## Supplementary Information

**Additional file 1.**

**Additional file 2:** **Table S1.** Cesarean delivery profiles of the study population using Robson classification. **Table S2.** Characteristics of group 5a women according to pre-pregancy BMI. **Table S3.** Association between maternal obesity and CS before labor for Robson group 5a *N* = 1380. **Table S4.** Association between maternal obesity and CS during labor for Robson group 5a *N* = 1304. **Table S5.** neonatal outcomes of twin pregnancies according to maternal pre-pregnancy BMI.

## Data Availability

Raw data including the database are available in the supplementary files.

## Abbreviations

BMI: Body mass index
CS: Cesarean section
GWG: Gestational weight gain
