# Supplementary material for: Use of the Robson classification to understand the increased risk of cesarean section in case of maternal obesity
Source: BMC Pregnancy Childbirth. 2020 Nov 26;20:738. doi: 10.1186/s12884-020-03410-z (PMC7690087; doi:10.1186/s12884-020-03410-z)
Supplement: Supplementary file 2 — Additional file 2: Table S1. Cesarean delivery profiles of the study population using Robson classification. Table S2. Characteristics of group 5a women according to pre-pregancy BMI. Table S3. Association between maternal obesity and CS before labor for Robson group 5a N = 1380. Table S4. Association between maternal obesity and CS during labor for Robson group 5a N = 1304. Table S5. neonatal outcomes of twin pregnancies according to maternal pre-pregnancy BMI. [file 12884_2020_3410_MOESM2_ESM.docx]

| ***Group**** | ***N CS in group*** | ***Total N in group*** | ***Group Size (%)1*** | ***Group CS rate (%)2*** | ***Absolute group contribution to overall CS rate (%)3*** | ***Relative group contribution to overall CS rate (%)4*** | ***Percentage of women with obesity*** |
| --- | --- | --- | --- | --- | --- | --- | --- |
| ***1*** | 347 | 3572 | 24.2 | 9.7 | 2.3 | 13.8 | 11.6 |
| ***2*** | 387 | 1370 | 9.3 | 28.2 | 2.6 | 15.4 | 8.0 |
| *2a (Induced)* | 317 | 1300 | 8.8 | 24.4 | 2.1 | 12.6 | 7.5 |
| *2b (Prelabor CS)* | 70 | 70 | 0.5 | 100.0 | 0.5 | 2.8 | 0.5 |
| ***3*** | 111 | 4853 | 32.8 | 2.3 | 0.8 | 4.4 | 30.4 |
| ***4*** | 103 | 1086 | 7.3 | 9.5 | 0.7 | 4.1 | 11.7 |
| *4a (Induced)* | 72 | 1055 | 7.1 | 6.8 | 0.5 | 2.9 | 11.4 |
| *4b (Prelabor CS)* | 31 | 31 | 0.2 | 100.0 | 0.2 | 1.2 | 0.4 |
| ***5*** | 702 | 1733 | 11.7 | 40.5 | 4.7 | 28.0 | 22.0 |
| *5.a (1 CS)* | 358 | 1380 | 9.3 | 25.9 | 2.4 | 14.3 | 15.6 |
| *5.b (> 1 CS)* | 344 | 353 | 2.4 | 97.5 | 2.3 | 13.7 | 6.4 |
| ***6*** | 167 | 301 | 2.0 | 55.5 | 1.1 | 6.7 | 1.0 |
| ***7*** | 226 | 402 | 2.7 | 56.2 | 1.5 | 9.0 | 4.5 |
| ***8*** | 204 | 582 | 3.9 | 35.1 | 1.4 | 8.1 | 3.5 |
| ***9*** | 30 | 30 | 0.2 | 100.0 | 0.2 | 1.2 | 0.3 |
| ***10*** | 231 | 859 | 5.8 | 26.9 | 1.6 | 9.2 | 6.9 |
| ***Total*** | 2508 | 14788 | 100.0% | 17.0 | 17.0 | 100.0 | 100.0 |

**Table S1: Cesarean delivery profiles of the study population using Robson classification.**

*See definitions in Table 2

**Table S2: Characteristics of group 5a women according to pre-pregancy BMI**

* High medical risk level at the beginning of pregnancy was defined as the presence of one or more of: history of cardiac disease, hypertension, diabetes, venous
thrombosis, pulmonary embolism, Graves’ disease, asthma, homozygous sickle cell anemia, thrombocytopenia, coagulation disorder, a rare or systemic disease,
nephropathy, HIV infection, pre-eclampsia, growth restriction, preterm delivery, fetal death or neonatal death.

** Defined as the occurrence of one or more of the following complications: gestational diabetes, gestational hypertension, pre-eclampsia, eclampsia, HELLP syndrome,
venous thrombosis, pulmonary embolism, severe sepsis, convulsions, diabetic ketoacidosis, coagulation disorder, cholestasis of pregnancy

*** Excessive total gestational weight gain defined as an intake of more than 9 kg for women with obesity and an intake of more than 15.9 kg for normal weight women.

|  | Normal weight | Obesity |  |
| --- | --- | --- | --- |
|  | 912 | 468 |  |
|  | N (%) | N (%) | p |
| **Maternal characteristics** |  |  |  |
| age (mean±sd) | 33.57±5.21 | 33.78±5.06 | 0.473 |
| age class |  |  | 0.063 |
| < 25 | 52 (5.7) | 13 (2.8) |  |
| [25.30[ | 178 (19.5) | 107 (22.9) |  |
| [30;35[ | 315 (34.5) | 160 (34.2) |  |
| [>= 35[ | 367 (40.2) | 188 (40.2) |  |
| Body mass index before pregnancy (kg/m²) (mean ± sd) | 21.76 (1.65) | 33.65 (3.80) | <0.001 |
| Smoker | 119 (13.3) | 45 (9.9) | 0.087 |
| Diabetes mellitus | 10 (1.1) | 20 (4.3) | <0.001 |
| Chronic hypertension | 11 (1.2) | 35 (7.5) | <0.001 |
| Bariatric surgery |  |  | <0.001 |
| Bypass | 2 (0.2) | 3 (0.6) |  |
| Sleeve gastrectomy | 1 (0.1) | 8 (1.7) |  |
| Gastric band | 1 (0.1) | 14 (3.0) |  |
| Hight medical risk level at the beginning of pregnancy* | 148 (16.2) | 154 (32.9) | <0.001 |
| **Pregnancy characteristics** |  |  |  |
| Complications of pregnancy** | 97 (10.6) | 138 (29.5) | <0.001 |
| Weight intake during pregnancy (mean±sd) | 12.46±5.48 | 7.89±6.82 | <0.001 |
| Excessive total GWG*** | 232 (25.7) | 211 (46.1) | <0.001 |
| Gestational diabetes requiring insulin | 21 (2.3) | 48 (10.3) | <0.001 |
| Gestational diabetes without insulin | 48 (5.3) | 54 (11.5) | <0.001 |
| In utero transfer | 2 (0.2) | 0 (0.0) | 0.79 |
| Premature rupture of membranes | 3 (0.3) | 3 (0.6) | 0.688 |
| Preterm labor | 17 (1.9) | 4 (0.9) | 0.223 |
| Gestational hypertension | 9 (1.0) | 22 (4.7) | <0.001 |
| Preeclampsia | 16 (1.8) | 22 (4.7) | 0.003 |
| Suspected small for gestational age | 16 (1.8) | 6 (1.3) | 0.663 |
| Cholestasis | 5 (0.5) | 6 (1.3) | 0.258 |
| **Delivery characteristics** |  |  |  |
| Gestational age at delivery |  |  |  |
| >=41 | 197 (21.6) | 110 (23.5) | 0.461 |
| Induction | 201 (22.0) | 158 (33.8) | <0.001 |
| Induction indication |  |  | <0.001 |
| Fetal | 55 (6.0) | 43 (9.2) | 0.04 |
| Maternal | 15 (1.6) | 20 (4.3) | 0.005 |
| Premature rupture of membranes | 83 (9.1) | 43 (9.2) | 1 |
| Post term | 27 (3.0) | 28 (6.0) | 0.01 |
| Gestational hypertension or preeclampsia | 20 (2.2) | 23 (4.9) | 0.009 |
| Non medical | 1 (0.1) | 1 (0.2) | 1 |
| Delivery mode |  |  | <0.001 |
| Vaginal delivery | 708 (77.6) | 314 (67.1) |  |
| CS before labor | 44 (4.8) | 32 (6.8) |  |
| CS during labor | 160 (17.5) | 122 (26.1) |  |
| Cesarean section indication |  |  |  |
| Abnormal fetal heart rate | 86 (9.4) | 64 (13.7) | 0.02 |
| Arrest of labor | 76 (8.3) | 62 (13.2) | 0.005 |
| Cesarean scar | 20 (2.2) | 12 (2.6) | 0.8 |
| Maternal | 10 (1.1) | 6 (1.3) | 0.97 |
| Fetal indication or placenta malposition | 12 (1.3) | 10 (2.1) | 0.35 |

**Table S3: Association between maternal obesity and CS before labor for Robson group 5a N = 1380**

Abbreviations : OR odds ratio, aOR adjusted odds ratio, 95% CI 95% confidence interval

*Logistic regression models including all variables in the column

** see definitions in table 1

|  | Cesarean section before labor versus trial of labor | |
| --- | --- | --- |
|  | OR [95% CI] | aOR* [95% CI] |
| Age > 35 | 0.91 [0.56-1.47] | 0.85 [0.52-1.37] |
| Obesity | 1.45 [0.90-2.31] | 1.26 [0.76-2.08] |
| Hight medical risk level at the beginning of pregnancy** | 0.87 [0.49-1.56] | 0.77 [0.43-1.40] |
| Complications of pregnancy** | 2.23 [1.34-3.73] | 2.17 [1.27-3.73] |

**Table S4 : Association between maternal obesity and CS during labor for Robson group 5a N = 1304**

Abbreviations : OR odds ratio, aOR adjusted odds ratio, 95% CI 95% confidence interval

*Logistic regression models including all variables in the column

** see definitions in table 1

|  | Cesarean section during labor versus vaginal delivery | |
| --- | --- | --- |
|  | OR [95% CI] | aOR* [95% CI] |
| Age > 35 | 1.08 [0.83-1.41] | 1.02 [0.77-1.34] |
| Obesity | 1.72 [1.31-2.25] | 1.43 [1.07-1.9] |
| Induction | 2.01 [1.52-2.67] | 1.82 [1.36-2.43] |
| Hight medical risk level at the beginning of pregnancy** | 1.31 [0.97-1.79] | 1.19 [0.86-1.63] |
| Complications of pregnancy** | 1.99 [1.44-2.75] | 1.60 [1.14-2.26] |

**Table S5: neonatal outcomes of twin pregnancies according to maternal pre-pregnancy BMI**

|  | Normal weight | Obesity | p |
| --- | --- | --- | --- |
|  | N=481 | N=105 |  |
|  | N (%) | N (%) |  |
| Birth weight J1 (grams) (mean±sd) | 2338±570 | 2410±663 | 0.258 |
| Birth weight J1 (grams) |  |  | 0.014 |
| [2500-3800[ | 203 (42.2) | 55 (52.4) |  |
| < 2500 | 278 (57.8) | 49 (46.7) |  |
| > 3800 | 0 (0.0) | 1 (1.0) |  |
| Birth weight J2 (grams) (mean±sd) | 2275±549 | 2387±676 | 0.073 |
| Birth weight J2 (grams) |  |  | 0.006 |
| [2500-3800[ | 182 (38.0) | 52 (50.0) |  |
| < 2500 | 297 (62.0) | 51 (49.0) |  |
| > 3800 | 0 (0.0) | 1 (1.0) |  |
| pH at umbilical cord J1 |  |  | 0.331 |
| pH < 7 | 0 | 0 |  |
| pH [7-7,1] | 5 (1.1) | 0 (0.0) |  |
| pH ]7.1-7.2[ | 17 (3.6) | 6 (5.9) |  |
| pH ≥ 7.2 | 449 (95.3) | 95 (94.1) |  |
| pH at umbilical cord J2 |  |  | 0.61 |
| pH < 7 | 2 (0.4) | 0 (0.0) |  |
| pH [7-7,1] | 4 (0.8) | 2 (2.0) |  |
| pH ]7.1-7.2[ | 17 (3.6) | 5 (4.9) |  |
| pH ≥ 7.2 | 448 (95.1) | 95 (93.1) |  |
| Apgar score < 7 at 5 minutes J1 | 5 (1.0) | 5 (4.8) | 0.024 |
| Apgar score < 7 at 5 minutes J2 | 12 (2.5) | 4 (3.8) | 0.669 |
| Transfer place J1 |  |  | 0.252 |
| Neonatal reanimation unit | 114 (23.8) | 16 (15.2) |  |
| Intensive care unit | 98 (20.4) | 26 (24.8) |  |
| Other specialized services | 1 (0.2) | 0 (0.0) |  |
| Transfer place J2 |  |  | 0.26 |
| Neonatal reanimation unit | 109 (22.7) | 16 (15.2) |  |
| Intensive care unit | 120 (25.0) | 32 (30.5) |  |
| Other specialized services | 3 (0.6) | 0 (0.0) |  |
